# Supplementary material for: The interactome of intact mitochondria by cross-linking mass spectrometry provides evidence for coexisting respiratory supercomplexes
Source: Mol Cell Proteomics. 2017 Dec 8;17(2):216–32. doi: 10.1074/mcp.RA117.000470 (PMC5795388; doi:10.1074/mcp.RA117.000470)
Supplement: Supplemental Data [file supp_RA117.000470_133922_0_supp_23978_fzffwf.zip › spectra_annotation/mito_spectra_annotation/18-1-9-1-16-1.pdf]

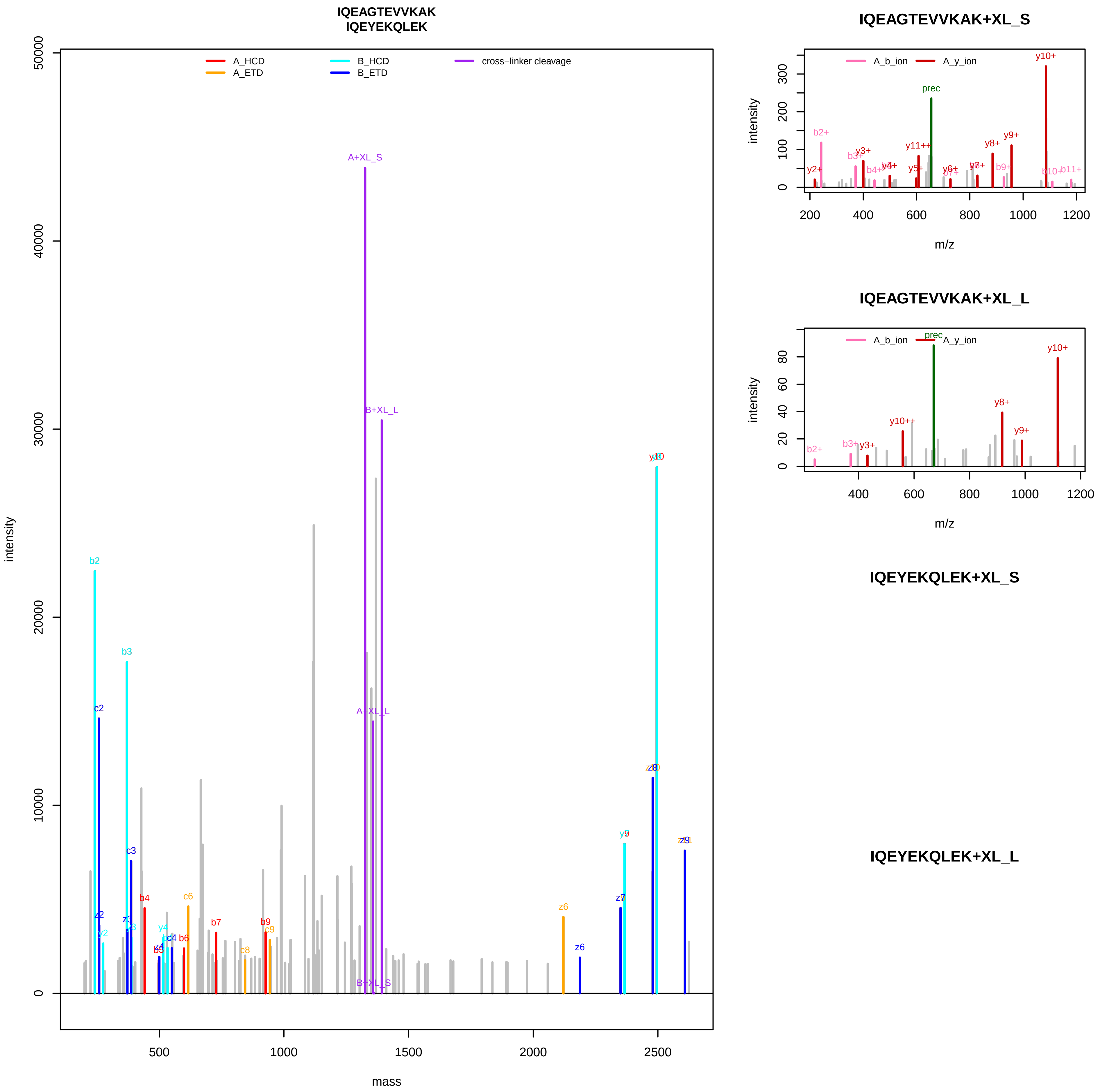
IQEAGTEVVKAK
IQEYEKQLEK
A_HCD
A_ETD
B_HCD
B_ETD
cross−linker cleavage
intensity
mass
A+XL_S
B+XL_L
A+XL_L
B+XL_S
b2
b3
c2
c3
b4
c6
b7
b9
c9
c8
b6
c4
z6
z7
z8
y9
z9
y10
IQEAGTEVVKAK+XL_S
A_b_ion
A_y_ion
prec
intensity
m/z
y10+
y11++
y9+
y8+
y3+
b2+
b3+
y2+
IQEAGTEVVKAK+XL_L
y10++
IQEYEKQLEK+XL_S
IQEYEKQLEK+XL_L
